# Unanswered Questions About Microscopic Hematuria With Tubulopathy

**DOI:** 10.1016/j.ekir.2026.106637

**Published:** 2026-05-30

**Authors:** Guy H. Neild, D. Deren Oygar, Ahmet Behlul, Salahi Ataçi, Meral Yükseliş, Simge Özadalı, Fezile Ozdemir, Hasan Hüseyin Kazan, Daniel P. Gale, Cemal Gurkan

**Affiliations:** 1 UCL Centre for Kidney and Bladder Health, University College, London, UK; 2 Nephrology Department, Nicosia State Hospital, Dr. Burhan Nalbantoglu General Hospital, Nicosia, Cyprus; 3 Dr. Fazıl Küçük Faculty of Medicine, Eastern Mediterranean University, Famagusta, Cyprus; 4 Turkish Cypriot DNA Laboratory, Committee on Missing Persons in Cyprus Turkish Cypriot Member Office, Nicosia, Cyprus; 5 Department of Medical Biology, Gulhane Faculty of Medicine, University of Health Sciences, Ankara, Türkiye

To the Editor:

We read with great interest that Riella *et al.*^1^ found pathogenic heterozygous variants of *COL4A3*, *COL4A4*, or *COL4A5* in only a third of their US cohort with clinical features of thin glomerular basement membrane glomerular disease.

Their results resonate with accumulating observations of kidney diseases in Cyprus. We have reported predicted-pathogenic *COL4A* variants in just 7 of 55 families (13%) with an apparent dominant inheritance pattern of chronic kidney disease.^2^ This concurs with another report from Cyprus in which only 28% of microscopic hematuria families carried pathogenic *COL4* variants.^3^

Reviewing the detailed clinical features in a Cyprus cohort of 20 families without overt *COL4* mutations, each with multiple affected members, we find that microscopic hematuria was detected at least once in 76% of affected individuals and hypertension was universal and easily controlled with 1 to 2 drugs. Little or no proteinuria (< 1 g/d) was typical in chronic kidney disease grades 1 to 3, and chronic kidney disease 5 occurred in a quarter of those aged >50 years at a median of 62.8 (range: 36−86) years. Therefore, these families have features of both glomerular and tubular disease and resemble so-called “hypertensive nephropathy.”

Furthermore, pathogenic variants in other (non-*COL4*) Mendelian glomerulopathy genes (e.g., *WT1*, *INF2*, *PAX2*, etc.) are also absent in these families. In addition, they do not carry mutations in autosomal dominant tubulopathy genes, including *UMOD* and *HNF1B*. However, approximately half of these affected family members carry 1 of 3 common missense variants (*COL4A4*: p.G545A and p. G999E, and *COL4A3*: p.G43R). Although these are not predicted to be overtly pathogenic, one can speculate that they somehow predispose to the phenotype; *COL4* genes are expressed in a subset of tubules, as well as in glomeruli.^4^ Other, yet-to-be defined, genomic factors may be operative, as well as modifying environmental “hits” such as diabetes mellitus, which is common in Cyprus.
